# Metabolic changes associated with adaptive resistance to daptomycin in *Streptococcus mitis*-*oralis*

**DOI:** 10.1186/s12866-020-01849-w

**Published:** 2020-06-15

**Authors:** Allison Parrett, Joseph M. Reed, Stewart G. Gardner, Nagendra N. Mishra, Arnold S. Bayer, Robert Powers, Greg A. Somerville

**Affiliations:** 1 Department of Chemistry, University of Nebraska-Lincoln, Lincoln, NE 68588-0304 USA; 2 School of Veterinary Medicine and Biomedical Sciences, University of Nebraska-Lincoln, Lincoln, NE 68588-0905 USA; 3 Present address: Chemical Testing Program, Wyoming Department of Health, Cheyenne, Wyoming 82002 USA; 4 Present address: Department of Biological Sciences, Emporia State University, Emporia, Kansas 66801 USA; 5 Division of Infectious Diseases, The Lundquist Institute at Harbor-UCLA Medical Center, Torrance, California 90502 USA; 6 David Geffen School of Medicine University of California Los Angeles, Los Angeles, California 90095 USA; 7 Nebraska Center for Integrated Biomolecular Communication, University of Nebraska-Lincoln, Lincoln, NE 68588-0304 USA

**Keywords:** Streptococcus, Metabolism, Antibiotic resistance, Daptomycin

## Abstract

**Background:**

Viridans group streptococci of the *Streptococcus mitis-oralis* subgroup are important endovascular pathogens. They can rapidly develop high-level and durable non-susceptibility to daptomycin both in vitro and in vivo upon exposure to daptomycin. Two consistent genetic adaptations associated with this phenotype (i.e., mutations in *cdsA* and *pgsA*) lead to the depletion of the phospholipids, phosphatidylglycerol and cardiolipin, from the bacterial membrane. Such alterations in phospholipid biosynthesis will modify carbon flow and change the bacterial metabolic status. To determine the metabolic differences between daptomycin-susceptible and non-susceptible bacteria, the physiology and metabolomes of *S. mitis-oralis* strains 351 (daptomycin-susceptible) and 351-D10 (daptomycin non-susceptible) were analyzed. *S. mitis-oralis* strain 351-D10 was made daptomycin non-susceptible through serial passage in the presence of daptomycin.

**Results:**

Daptomycin non-susceptible *S. mitis-oralis* had significant alterations in glucose catabolism and a re-balancing of the redox status through amino acid biosynthesis relative to daptomycin susceptible *S. mitis-oralis*. These changes were accompanied by a reduced capacity to generate biomass, creating a fitness cost in exchange for daptomycin non-susceptibility.

**Conclusions:**

*S. mitis-oralis* metabolism is altered in daptomycin non-susceptible bacteria relative to the daptomycin susceptible parent strain. As demonstrated in *Staphylococcus aureus*, inhibiting the metabolic changes that facilitate the transition from a daptomycin susceptible state to a non-susceptible one, inhibits daptomycin non-susceptibility. By preventing these metabolic adaptations in *S. mitis-oralis*, it should be possible to deter the formation of daptomycin non-susceptibility*.*

## Background

*Staphylococcus aureus* is the most common cause of infective endocarditis (IE) in the industrialized world [1, 2]. The viridans group streptococci are the second leading IE pathogen world-wide, and the most frequent etiology of IE in developing countries. Among the viridans group streptococci, the *S. mitis-oralis* subgroup (i.e., *S. mitis*, *S. oralis*, *S. gordonii* and *S. parasanguinis*) are the predominant IE etiologies. This sub-group is therapeutically problematic, as between 10 and 40% of strains exhibit resistance to penicillins and/or cephalosporins, including ceftriaxone [3, 4]. For this reason, the majority of research into *S. mitis-oralis* antibiotic resistance to-date relates to the genetic determinants of penicillin resistance (e.g., [5]).

In addition to being multi-β-lactam-resistant, *S. mitis-oralis* can also be vancomycin-tolerant [6], which increases the use of daptomycin in treating infections caused by such bacteria. Importantly, daptomycin non-susceptibility arises rapidly both in vitro and in vivo (e.g., during the treatment experimental infective endocarditis [7]), causing great concern this could occur in humans undergoing daptomycin therapy for streptococcal IE. In *S. mitis-oralis*, daptomycin non-susceptibility is associated with mutations in *cdsA* and *pgsA* [8, 9], genes involved in biosynthesis of membrane phospholipids [10]. Specifically, these mutations in daptomycin non-susceptible *S. mitis-oralis* strains result in the loss of phosphotidylglycerol and cardiolipin from the membrane.

Although these studies have been critical for understanding the genetic perturbations that facilitate *S. mitis-oralis* non-susceptibility to daptomycin, the physiologic and metabolic modifications associated with adaptive resistance to daptomycin are unknown. In *S. aureus*, daptomycin non-susceptibility has been metabolically linked to decreased tricarboxylic acid (TCA) cycle activity, as well as increased nucleotide synthesis and carbon flow to pathways important for wall teichoic acid and peptidoglycan biosynthesis [11]. Because *S. mitis-oralis* lacks a TCA cycle and grows best in a reduced oxygen environment, it is likely the metabolic adaptations underpinning daptomycin non-susceptibility are different from those found in *S. aureus*. To determine the metabolic changes associated with adaptive resistance to daptomycin in *S. mitis-oralis*, the metabolomes of daptomycin-susceptible strain 351 and it’s in vitro-derived daptomycin non-susceptible variant, 351-D10, were analyzed using nuclear magnetic resonance (NMR) spectroscopy.

## Results

### Daptomycin non-susceptibility alters growth of *S. mitis*

**Fig. 1:**
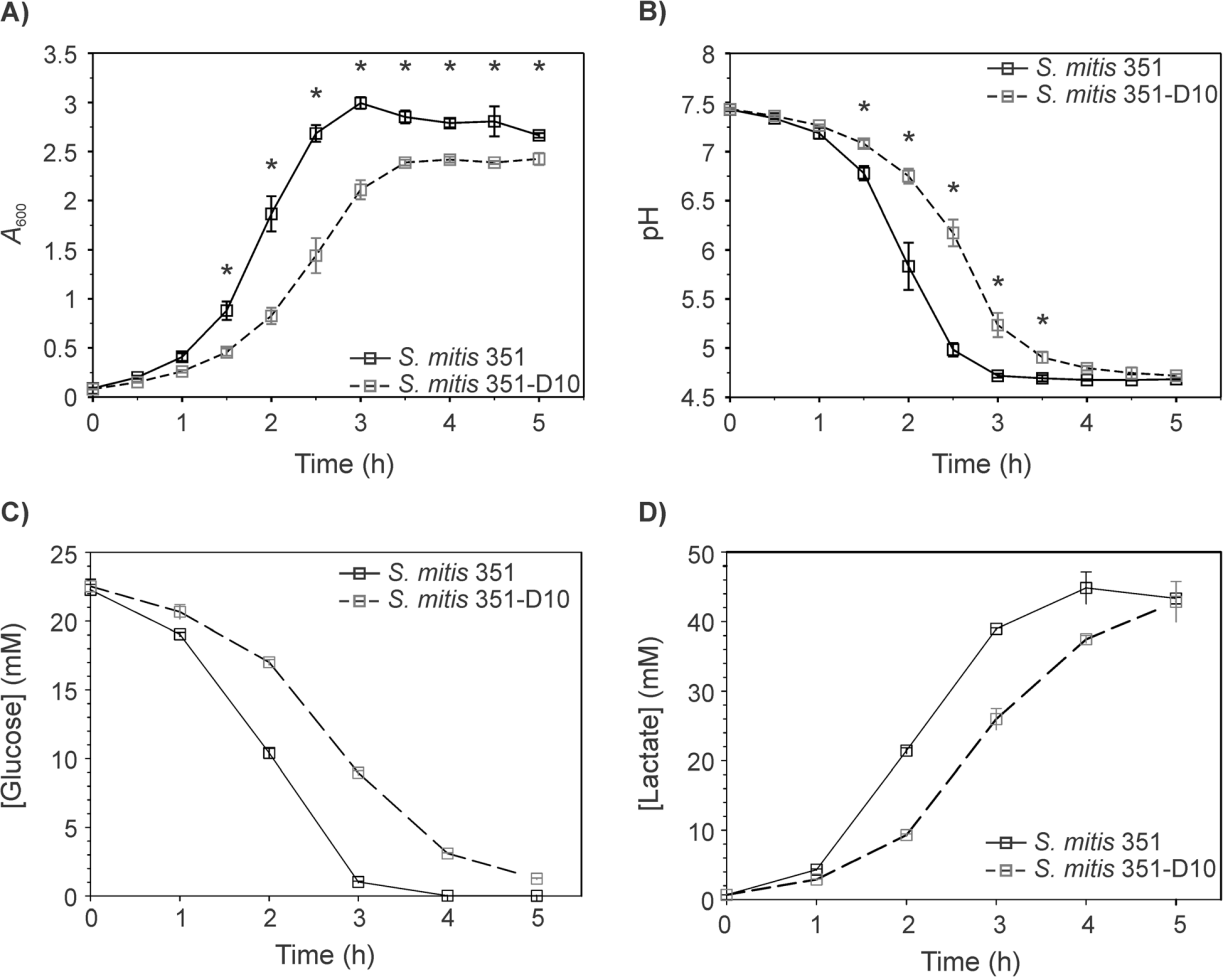
Growth characteristics of *S. mitis-oralis* strains 351 (black symbols, solid line) and 351-D10 (grey symbols, dashed line) cultivated in BHI with 2 g/L of supplemental glucose. The *A*_600_ (**a**) and pH (**b**) profiles were assessed every 30 min. Glucose depletion (**c**) and lactate accumulation (**d**) in the cultivation media were assessed hourly. Data are representative of the mean of experiments performed in biological triplicate, with error bars representing the standard deviation of the mean. Statistical differences in *A*_600_ and pH for strain 351 relative to strain 351-D10 was assessed by ANOVA. A statistically significant difference (*p* ≤ 0.01) is represented with an (*)

The transition of *S. mitis-oralis* strain 351 from a daptomycin-susceptible state to a non-susceptible state (strain 351-D10) during serial passage was accompanied by a decreased growth rate (351 and 351-D10; 29 ± 1 min and 39 ± 1.5 min, respectively) and a decreased growth yield/biomass (Fig. 1a and Supplemental Fig. 1). This altered growth phenotype was reflected in the significant differences in the rates of cultivation media acidification (Fig. 1b). As expected, the growth changes also decreased glucose depletion from the medium containing strain 351-D10 relative to that of strain 351 (Fig. 1c and Supplemental Fig. 2). Importantly, the total amount of glucose consumed by both strains was equivalent, yet the difference in biomass between the two strains was significant, suggesting that growth alone did not account for all the differences in glucose consumption. The glycolytic end-product of glucose is pyruvate, which, in *S. mitis-oralis*, is predominantly catabolized by the membrane-associated lactate dehydrogenase [12]. The lactic acid produced by lactate dehydrogenase accumulated in the cultivation media, which caused the pH of the media to decrease over time (Fig. 1b and d). Similar to the depletion of glucose from the media, the accumulation of lactate in the media was largely dependent on bacterial growth (Fig. 1d and Supplemental Fig. 2). Taken together, the transition of *S. mitis-oralis* to a daptomycin non-susceptible state was accompanied by significant fitness changes resulting from impaired growth, and, likely, altered metabolism.

### The transition to a daptomycin non-susceptible state significantly alters metabolism

**Fig. 2:**
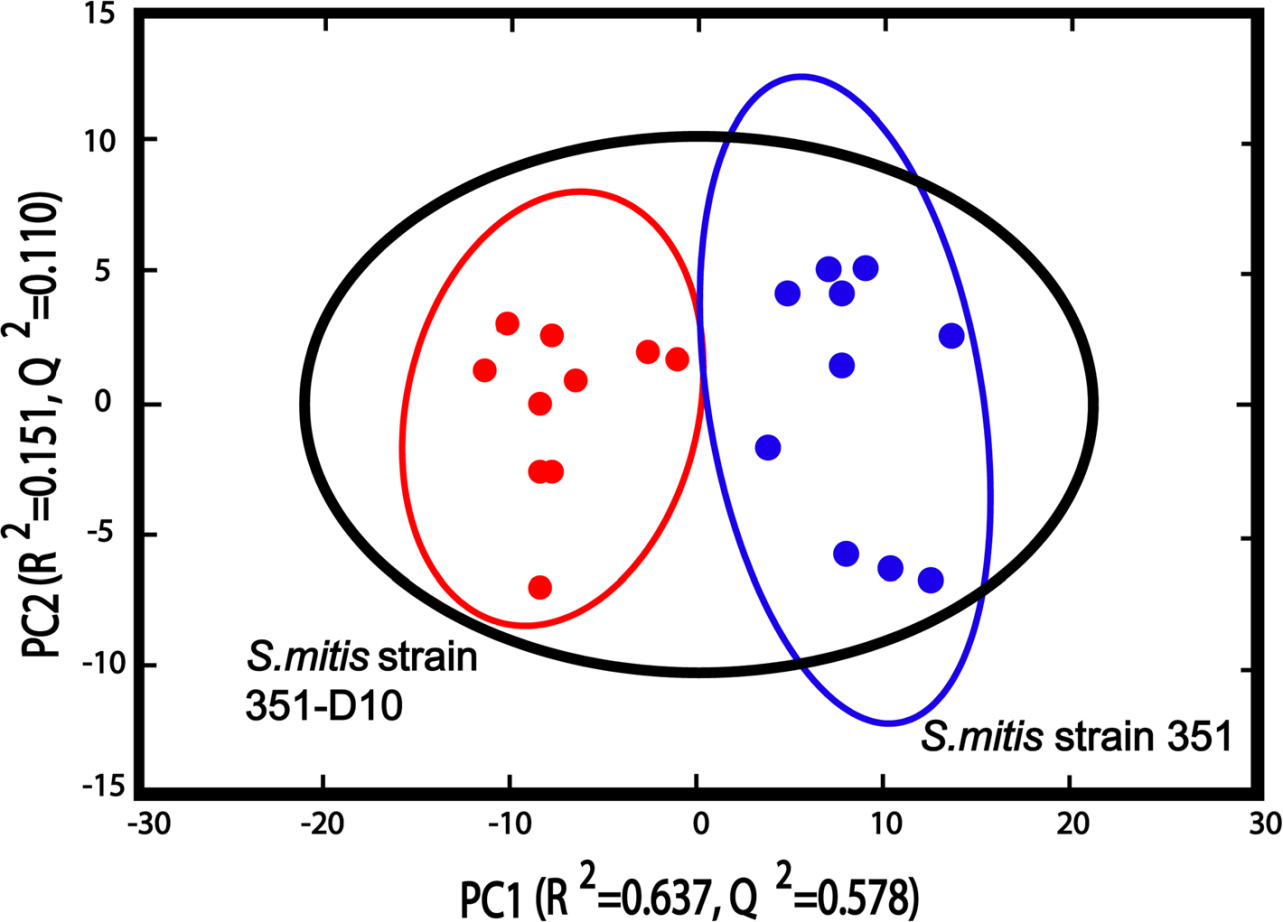
The PCA scores plot was generated from 1D ^1^H NMR spectra collected using cell-free lysates of *S. mitis-oralis* strains 351 (blue) and 351-D10 (red) cultivated in BHI with 2 g/L of supplemental ^13^C-glucose and harvested at 2 h (351) or 2 h 45 min (351-D10). The R^2^ and Q^2^ are 0.931 and 0.781 respectively for the PCA model

The growth profiling of *S. mitis-oralis* strains 351 and 351-D10 suggests metabolism was altered during the transition to a daptomycin non-susceptible state. To assess the extent of metabolic alterations, ten independent replicates of cell-free lysates from strains 351 and 351-D10 cultivated with ^13^C-glucose were harvested, the 1D ^1^H NMR spectra were collected, and the spectra were analyzed by PCA (Fig. 2). To normalize the metabolomic samples for the dissimilar growth kinetics of the two strains, and to ensure the metabolomes represented equivalent growth phases, bacteria were harvested at different cultivation times (i.e., 2 h for strain 351 and 2 h 45 min for strain 351-D10). As expected, the PCA scores plot revealed that daptomycin susceptible and non-susceptible strains each formed well-separated clusters (Fig. 2), confirming that significant metabolic differences arose during the transition to daptomycin non-susceptibility.

**Fig. 3:**
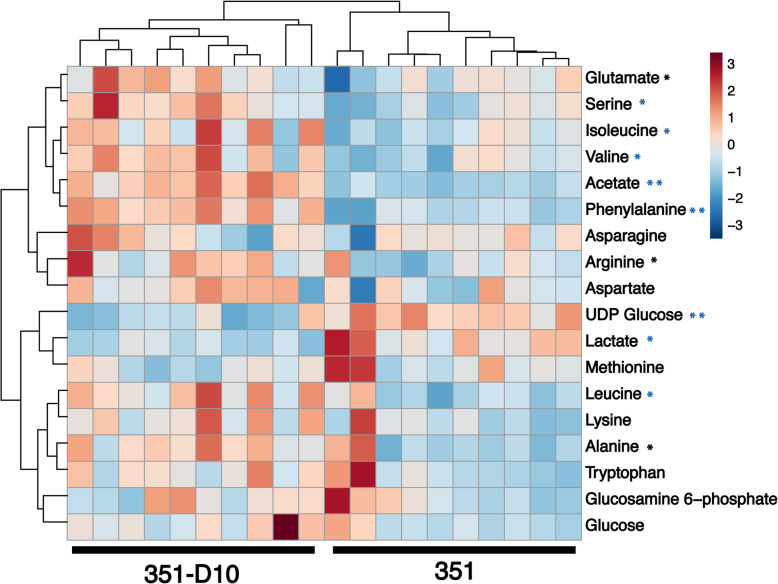
Heatmap with hierarchal clustering of metabolomic differences between *S. mitis-oralis* strains 351 and 351-D10. The map was generated using 2D ^1^H ^13^C HSQC NMR spectra with normalized peak intensities. Statistical significance was calculated using Student’s t-test (* *p* < 0.05 and ** *p* < 0.001) or Student’s t-test with Benjamini-Hochberg correction for multiple hypothesis (the asterisk in blue)

**Fig. 4:**
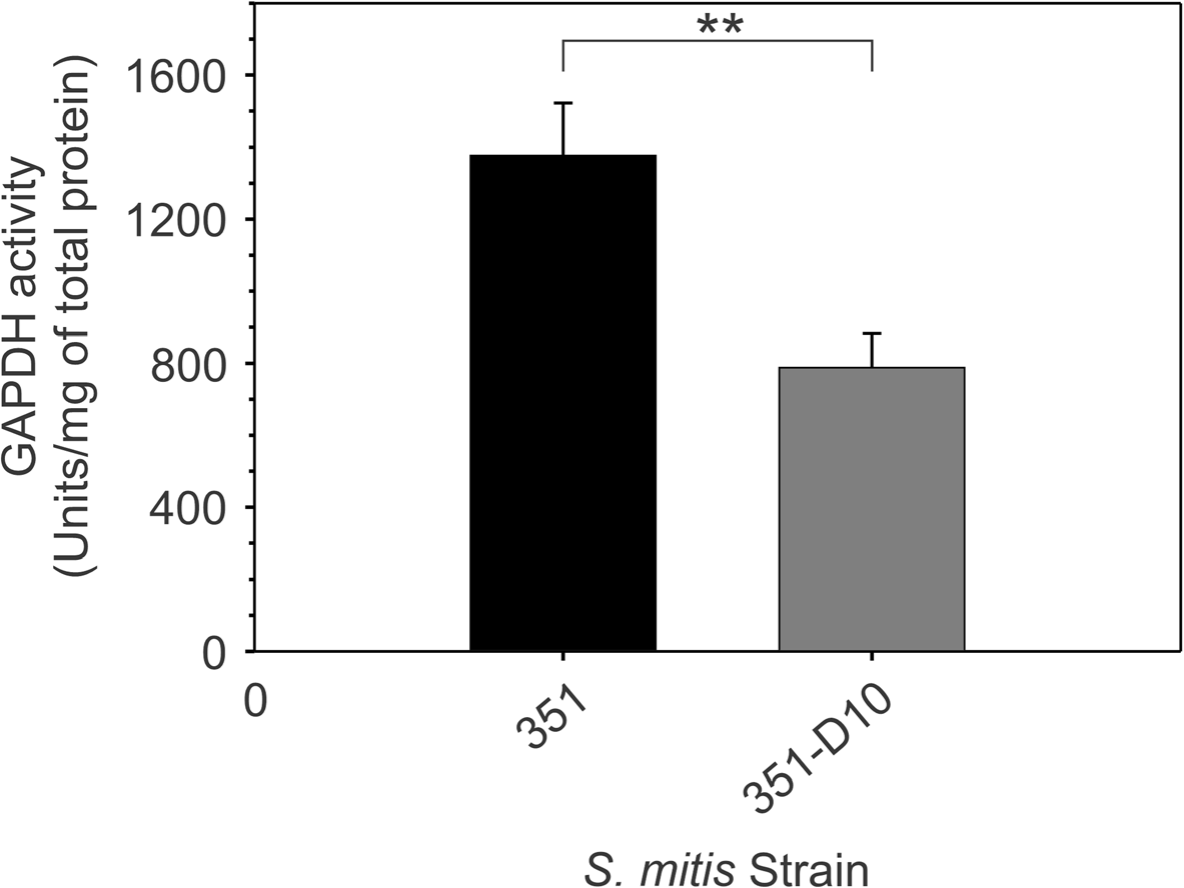
GAPDH activity in *S. mitis-oralis* strain 351-D10 is significantly reduced relative to strain 351. GAPDH activity was measured in *S. mitis-oralis* cell lysates from cultures cultivated in BHI and harvested after 1.5 h (351) or 2.5 h (351-D10). The data represent the mean and standard deviation from the mean of 3 biological replicates. Statistical significance (**) was assessed using Student’s t-test (*p* ≤ 0.05)

The PCA scores plot established significant metabolic differences existed between daptomycin-susceptible and non-susceptible *S. mitis-oralis* strains (Fig. 2), and the growth profiles indicated these differences were likely related to the enzymatic processing of glucose (Fig. 1c and d). To assess the differences in glucose catabolism and downstream pathways between strains 351 and 351-D10, the bacterial cell-free lysates used in the PCA analysis were analyzed by collecting 2D ^1^H- ^13^C HSQC NMR (Fig. 3 and Supplemental Fig. 3). Consistent with the growth profiles, *S. mitis-oralis* strain 351-D10 had significantly decreased intracellular levels of activated glucose (i.e., UDP-glucose) and lactic acid relative to strain 351 (Fig. 3). In contrast, strain 351-D10 had a significantly greater level of acetic acid, indicating that pyruvate generated from glycolysis was used for substrate-level phosphorylation as opposed to almost exclusively being used for the oxidation of NADH via lactate dehydrogenase. This difference was consistent with decreased GAPDH activity in strain 351-D10 (Fig. 4). Decreased GAPDH activity would slow the accumulation of NADH, but also decrease glycolytic ATP synthesis. As mentioned, strain 351-D10 likely offsets the decrease in glycolytic ATP synthesis through substrate phosphorylation via the phosphotransacetylase/acetate kinase pathway (Fig. 3).

## Discussion

Most studies of antibiotic resistance/non-susceptibility focus on genetic, transcriptional, and occasionally proteomic analyses [5, 8, 10]. These studies can provide valuable information; however, they describe changes that are predicted to occur (e.g., gene transcription does not necessarily equate with protein translation or activity). In contrast, metabolomics provides insight into cellular changes that have actually occurred and are likely to contribute to antibiotic non-susceptibility. Because metabolic pathways are finite in number, it is possible to ‘work backwards’ from the profile of altered metabolites and determine where the alterations arose.

In *S. aureus*, daptomycin non-susceptibility can be mediated by genetic changes in *mprF, walKR, rpoB*, and *rpoC* [13–16], changes in cell membrane composition [17, 18], and/or metabolic adaptations [11]. The metabolic adaptations in *S. aureus* cause a redirection of carbon flow from the TCA cycle into the pentose phosphate pathway. This redirection of carbon increases the availability of essential intermediates for the biosynthesis of peptidoglycan, wall teichoic acids, and nucleosides/nucleotides. These metabolic changes pre-adapt daptomycin non-susceptible *S. aureus* for daptomycin challenge. In other words, daptomycin challenge causes only minimal metabolic perturbations, which minimizes the fitness cost of daptomycin non-susceptibility [11]. The more likely reason for the minimal metabolic changes and low fitness cost associated with daptomycin non-susceptibility in *S. aureus* is due to its relatively robust metabolic capabilities, including the full complement of central metabolism pathways [19, 20]. In contrast, *S. mitis-oralis* lacks the central metabolism TCA cycle, creating metabolic deficiencies that require supplementation from the medium or a host. Alterations in the more modest metabolic capabilities of *S. mitis-oralis* would be consistent with profound growth differences between the susceptible and non-susceptible strains (Fig. 1).

**Fig. 5:**
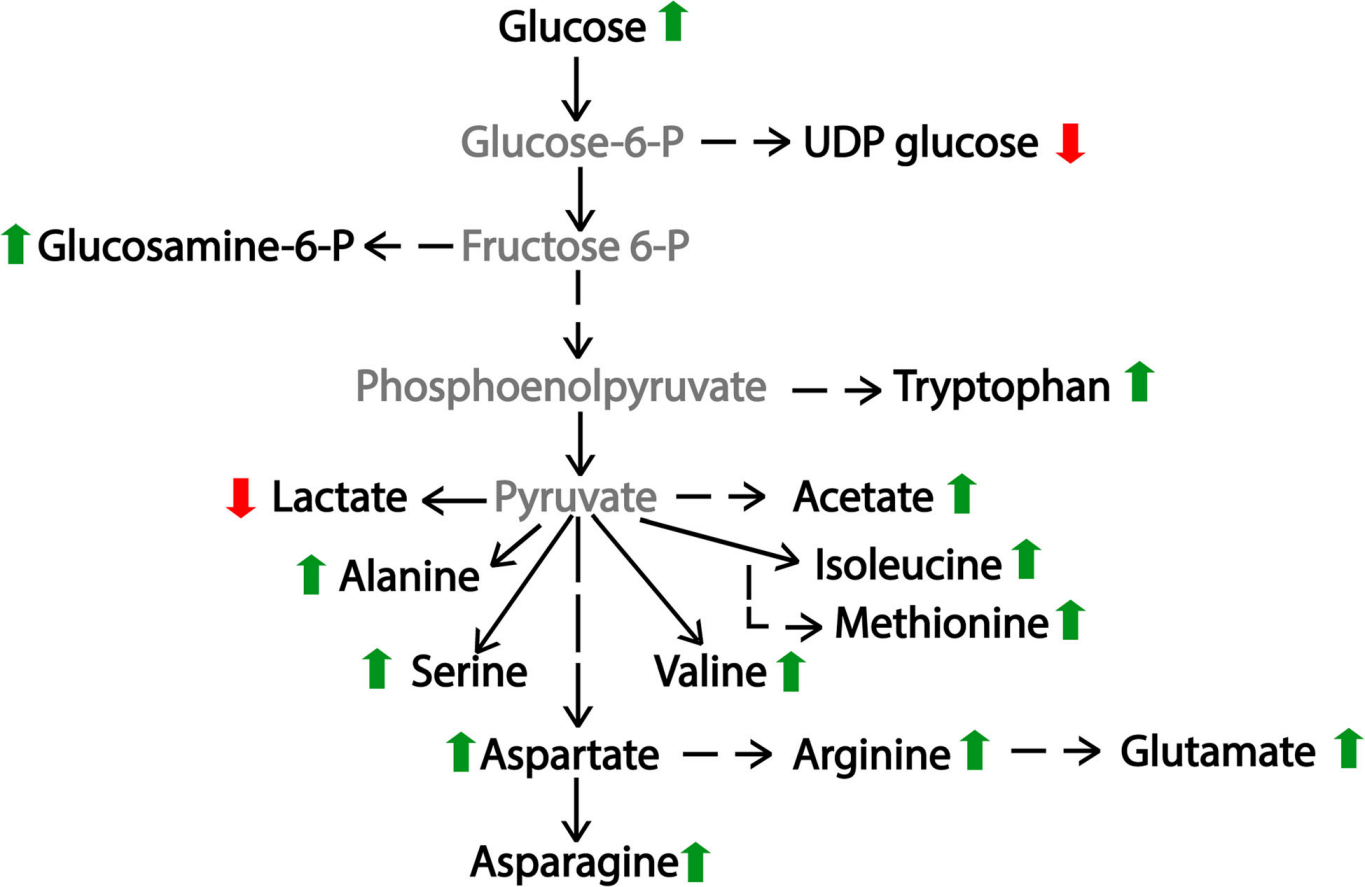
Summary of metabolic changes between *S. mitis-oralis* strains 351 and 351-D10. Green upward arrows represent a metabolite whose concentration is increased in strain 351-D10 relative to those in strain 351. Red downward arrows represent metabolites that are decreased in strain 351-D10 relative to that in strain 351. Metabolites in gray represent intermediates that were not observed in the NMR spectra, but were inferred from secondary metabolites

The growth differences between daptomycin susceptible and non-susceptible *S. mitis-oralis* strains are indicative of significant metabolic changes. Metabolomics revealed these growth differences coincided with significant metabolic changes (Fig. 2). Most importantly, is an alteration in how glucose is catabolized (Figs. 1, 3, and 4). For a lactic acid bacterium, alterations in glycolysis and/or lactate dehydrogenase activity creates the problem of how to produce ATP and maintain the redox status. Generating ATP by substrate-level phosphorylation has the advantage of not reducing NAD^+^, which decreases the burden on lactate dehydrogenase to balance the redox state, although it does not eliminate the need to oxidize dinucleotides. In addition to differences in ATP generation, the altered/delayed glucose catabolism allows for the diversion of carbon into other metabolic pathways (Figs. 3 and 5), which can enable metabolic adaptations that overcome altered glucose utilization. In contrast to *S. aureus*, *S. mitis* did not shunt carbon into the oxidative branch of the pentose phosphate pathway, which would generate NADPH. This latter point further indicates that these metabolic changes are driven by the bacterial redox status.

In daptomycin non-susceptible *S. mitis-oralis*, the metabolomics data revealed that glucose carbons were more slowly catabolized through glycolysis and lactate dehydrogenase relative to the daptomycin-susceptible strain (Figs. 1 and 3). As the primary function of lactate dehydrogenase is maintaining redox balance, it is likely a dysregulation in redox status contributed to the slower growth of strain 351-D10. One possibility for maintaining the redox status is by funneling carbon through amino acid biosynthetic pathways that oxidize more dinucleotides (e.g., branched chain amino acids, glutamate, and alanine) than they reduce (e.g., serine) (Fig. 5). Funneling more carbon through amino acid synthesis can be done in such a way as to facilitate the oxidation of dinucleotides, but the ATP deficit created by decreasing carbon flow through glycolysis also needs to be offset. The increase in acetic acid strongly suggests the ATP deficit is balanced by substrate-level phosphorylation through the phosphotransacetylase/acetate kinase pathway. Taken together, these data suggest the transition of *S. mitis-oralis* to a daptomycin non-susceptible state is accompanied by changes in redox balance, ATP synthesis, and carbon utilization. The extent to which these metabolic changes confer daptomycin non-susceptibility, or are a consequence of mutations arising in phospholipid biosynthetic genes, remains to be determined. In addition, the contribution of each daptomycin-induced mutation to the metabolic changes remains to be determined. Lastly, an important unanswered question is whether these metabolic changes in daptomycin-non-susceptible *S. mitis-oralis* strains be exploited to prevent or overcome this phenotype, as demonstrated in daptomycin-non-susceptible *S. aureus* [21, 22]? On this point, metabolic adaptations that facilitate non-susceptibility to vancomycin and daptomycin have successfully been targeted in *S. aureus* to prevent the development of non-susceptibility and to re-sensitize non-susceptible bacteria to these antibiotics [21, 22]. This strategy has relied on using inhibitors of metabolic pathways important for non-susceptibility such that enzymatic activity is reduced to the point where it becomes detrimental to the development or maintenance of non-susceptibility. Similar success with *S. mitis-oralis* will likely target amino acid biosynthesis and/or glycolysis, but this work is on-going.

## Conclusions

Significant metabolic changes accompany the development of a daptomycin non-susceptible state in *S. mitis*. These changes alter the bacterial ability to utilize glucose. This is a significant observation because *S. mitis*, like most lactic acid bacteria, is heavily dependent upon glycolysis and lactate dehydrogenase to balance the redox state and provide energy and biosynthetic intermediates. Because these changes occur in an essential metabolic pathway, it should be possible to target glycolysis using drugs that increase stress on this pathway (e.g., oxamic acid) and prevent the development of daptomycin non-susceptibility and to re-sensitize non-susceptible bacteria to daptomycin.

## Methods

### Bacterial strains and cultivation conditions

*S. mitis-oralis* daptomycin-susceptible strain 351 (daptomycin MIC = 0.5 μg/ml) and its daptomycin non-susceptible derivative, 351-D10 (daptomycin MIC > 256 μg/ml), were used for metabolomic analysis [8]. *S. mitis-oralis* strain 351 was isolated from a patient with endocarditis [7] and the derivative strain 351-D10 was generated by in vitro serial passage of strain 351 for 10 days in medium containing 20 μg/ml of daptomycin [8]. Whole-genome sequencing of the two strains revealed that strain 351-D10 had accrued mutations at seven loci; specifically, orfs sm351–26 (*cdsA*), sm351–42 (*rpoB*), sm351–129 (*fni*), sm351–251 (unknown), sm351–669 (*pepT*), sm351–1076 (*rbn*), and sm351–1167 (*clpX*). *S. mitis* strains were cultivated in Bacto Brain Heart Infusion medium (BHI), BHI + 2 g/L D-Glucose (BHI + glucose), BHI + 2 g/L ^13^C-D-Glucose (BHI + ^13^C-glucose), or on blood agar (TSA with Sheep Blood). Bacterial pre-cultures were inoculated 1:100 from overnight cultures into 50 mL of BHI in a 50 mL conical tube and incubated at 37 °C, without aeration, for 4 h. These exponential growth phase pre-cultures were centrifuged for 5 min at 4500 rpm (3460.7 X g) at 22 °C and suspended in 2–3 mL of medium. Primary cultures were inoculated into 50 mL pre-warmed BHI, BHI + 2 g/L D-Glucose, or BHI + 2 g/L ^13^C-Glucose in a 50 mL conical tube, at an absorbance at 600 nm (*A*_600_) of 0.08, and incubated at 37 °C. The cultures were mixed by inversion every 30 min prior to sampling. The *A*_600_ and pH were recorded every 30 min for 5 h.

### Determination of glucose and lactate concentrations in cultivation media

Cultivation media were harvested hourly (1 mL) by centrifugation at 13,200 rpm (16,168 x g), for 5 min at 4 °C. The cell-free media were transferred to 1.5 mL microcentrifuge tubes, flash frozen in liquid nitrogen, and stored at − 20 °C until use. Glucose and lactate concentrations in the culture media were determined from three independent cultures with kits purchased from R-Biopharm (Glucose test kit, #10716251035 and L-Lactic acid test kit, #10139084035).

### Glyceraldehyde 3-phosphate dehydrogenase (GAPDH) activity assay

Due to differences in growth, bacteria were cultivated in BHI for 1.5 h (strain 351) or 2.5 h (strain 351-D10) and harvested by centrifugation at 5000 rpm (4272.5 x g) for 5 min at 4 °C. Supernatants were discarded and the cell pellets were suspended in 750 μL GAPDH assay buffer (BioVision, Inc) and lysed in lysing matrix B tubes using a FastPrep instrument. The lysates were centrifuged at 13,200 rpm (16,168 x g), for 5 min at 4 °C. Supernatants were transferred to pre-chilled 1.5 mL microcentrifuge tubes. GAPDH activity was determined from three independent cultures according to the manufacture’s instruction. Protein concentrations were determined using the Modified Lowry Protein Assay (Fisher Scientific). The harvest times and corresponding *A*_600_ units were different from those used in metabolomics experiments because excess glucose was not added to the cultivation medium for enzyme assays.

### NMR sample preparation

The preferred process for performing ^13^C-glucose metabolomics experiments is to use a glucose-free medium and add labelled glucose at a defined concentration. Unfortunately, glucose-free BHI was unavailable. Adding 2 g/L of ^13^C-glucose into BHI containing unlabeled glucose (2 g/L) meant that only 50% of the glucose was labeled. This limitation required the doubling of the number of bacteria harvested. Harvest times were chosen based on A_600_ numbers that gave maximal biomass, when most of the glucose was catabolized, but not when the bacteria were in the stationary phase. For these reasons, bacteria were harvested at two different times when the bacterial biomass was equivalent. Bacteria cultivated in BHI + ^13^C-glucose were grown for 2 h (351) or 2 h 45 min (351-D10) and harvested by centrifugation at 4500 rpm (3460.7 x g) for 5 min at 0 °C. Bacterial cell pellets were washed with ice cold ultrapure H_2_O (Milli-Q) and quenched in liquid nitrogen. Cells were placed on ice to thaw for 5 min and the cell pellets were suspended in 1 mL of ice cold ultrapure H_2_O. The *A*_600_ of the bacterial suspension was determined and 60 *A*_600_ units were diluted into 1 mL of ultrapure H_2_O. The samples were transferred to lysing matrix B tubes (MP Biomedicals) and lysed for 40 s using a FastPrep instrument at a speed setting of 6, then placed on ice for 5 min. The lysing matrix B tubes were centrifuged at 13,200 rpm (16,168 x g) for 5 min at 0 °C, and the supernatants were transferred to 2 mL microcentrifuge tubes pre-cooled to 0 °C. Ice-cold H_2_O (1 mL) was added to each lysing matrix B tube, vortexed, and the samples were lysed a second time. The lysing matrix B tubes were centrifuged for 5 min at 0 °C, and the supernatants were pooled. The pooled cell-free lysates were clarified by centrifugation at 13,200 rpm for 1 min at 0 °C. Approximately 1.3 mL of the cell-free lysate from each sample was transferred to a pre-chilled 1.5 mL microcentrifuge tube. 1 mL of each cell-free lysate was flash frozen in liquid nitrogen and stored at − 80 °C prior to lyophilization for NMR analysis. The remaining cell-free lysate, approximately 300 μL, was used to determine protein concentrations (for normalization) with a modified Lowry protein assay (Fisher Scientific). Lyophilized frozen cell-free lysates were, suspended in 190 μL of 50 mM phosphate buffer (pH = 7.2, uncorrected) in D_2_O, and 50 μM of 3-(trimethylsilyl)propionic-2,2,3,3-d_4_ acid sodium salt (TMSP) was added as a chemical shift reference. The samples were centrifuged for 20 min at 13,200 rpm and 0 °C to remove any remaining cell debris. The supernatants were transferred to 3 mm NMR tubes for data collection.

### NMR data collection and analysis

NMR spectra were collected at 298 K using a Bruker AVANCE III-HD 700 MHz spectrometer equipped with a 5 mm quadruple resonance QCI-P cryoprobe (^1^H, ^13^C, ^15^N, and ^31^P) with z-axis gradients. A SampleJet sample changer, an automatic tuning and matching accessory, and ICON-NMR software were used to automate the NMR data collection. The 1D ^1^H NMR spectra were collected with 32 K data points, 128 scans, 16 dummy scans, a relaxation delay of 1.5 s, and a spectral width of 11,160 Hz. The 1D ^1^H NMR spectra were collected using an excitation sculpting pulse sequence to suppress the water resonance [23]. The 2D ^1^H- ^13^C HSQC spectra were collected using non-uniform sampling (NUS) at a 25% sampling sparsity [24]. The 2D ^1^H- ^13^C HSQC spectra were acquired with 32 scans, 16 dummy scans, and a 1.5 s relaxation delay. The spectra were collected with 2 K data points and a spectral width of 11,160 Hz in the direct dimension, and 1 K data points and a spectral width of 29,059 Hz in the indirect dimension.

### NMR data processing and analysis

Our MVAPACK software suite (http://bionmr.unl.edu/mvapack.php) was used to process and analyze the 1D ^1^H NMR spectra [25]. The 1D ^1^H NMR spectra were processed with a single zero-fill and a 0.3 Hz line-broadening, and then Fourier transformed and automatically phased-corrected [26]. The 1D ^1^H NMR spectra were normalized with standard normal variate normalization, aligned using icoshift [27], and then referenced to the TMSP peak at 0 ppm. Noise regions were automatically removed from the spectra and the residual water signal (4.6–4.8 ppm) was manually removed. The 1D ^1^H NMR spectra were binned using adaptive intelligent binning [28] and scaled using unit variance (UV) scaling. The resulting data matrix was then used to create a principle component analysis (PCA) model.

NMRpipe [29] was used to process the 2D ^1^H-^13^C HSQC data set and NMRViewJ [30] was used to analyze the spectra and assign the metabolites. The Human Metabolomic Database (HMDB) (http://www.hmdb.ca/) was used to assign metabolites by matching reference chemical shifts from the database to the experimental spectra using a peak-error tolerance of 0.08 ppm and 0.25 ppm for ^1^H and ^13^C chemical shifts, respectively [31]. A data matrix consisting of relative metabolite peak intensities (rows) and biological replicates (columns) was produced from the 2D ^1^H-^13^C HSQC data set [32, 33]. The data matrix was normalized using probabilistic quotient (PQ) normalization [32, 33]. BioCyc (https://biocyc.org) was used to identify the dysregulated *S. mitis* metabolic pathways based on the observed metabolite changes [34].

### Statistical analysis

Growth profiles and assays were performed in triplicate. Statistical differences in growth profiles were assessed by ANOVA, where a *p* ≤ 0.01 was defined as statistically significant. Statistical differences in assays were assessed by Student’s t-test, where a *p* ≤ 0.05 was defined as statistically significant.

A pair-wise Student’s t-test was used to determine if strain-dependent alterations in metabolite concentrations were statistically significant. The *p*-values were corrected using the Benjamini-Hochberg procedure to minimize the false discovery rate for multiple hypothesis testing [35]. Metabolite concentration changes were determined to be statistically significant by an FDR corrected *p*-value < 0.05. In order to visualize the differences between *S. mitis* strains, MetaboAnalyst (https://www.metaboanalyst.ca) was used to generate a heatmap with hierarchical clustering from the 2D ^1^H-^13^C HSQC NMR peak intensities [36]. The data matrix of NMR peak intensities was scaled along each row using standard normal variate (SNV).

## Supplementary information

**Additional file 1: Figure S1.** A semi-log plot of bacterial growth with supplemental glucose.

**Additional file 2: Figure S2.** Glucose depletion and lactate accumulation as a function of growth

**Additional file 3: Figure S3.** Representative 2D ^1^H ^13^C HSQC NMR spectra for *S. mitis* strains 351 and 351-D10

## Data Availability

The NMR metabolomics data can be found at https://figshare.com/s/6a04fdcb4ac4546db276. All other data generated or analyzed during this study are included in this published article.

## Abbreviations

IE: Infective endocarditis
NMR: Nuclear magnetic resonance
BHI: Brain heart infusion medium
GAPDH: Glyceraldehyde 3-phosphate dehydrogenase
HSQC: Heteronuclear single quantum coherence
