# Supplementary figures and images for: Metabolic changes associated with adaptive resistance to daptomycin in Streptococcus mitis-oralis
Source: BMC Microbiol. 2020 Jun 15;20:162. doi: 10.1186/s12866-020-01849-w (PMC7296729; doi:10.1186/s12866-020-01849-w)

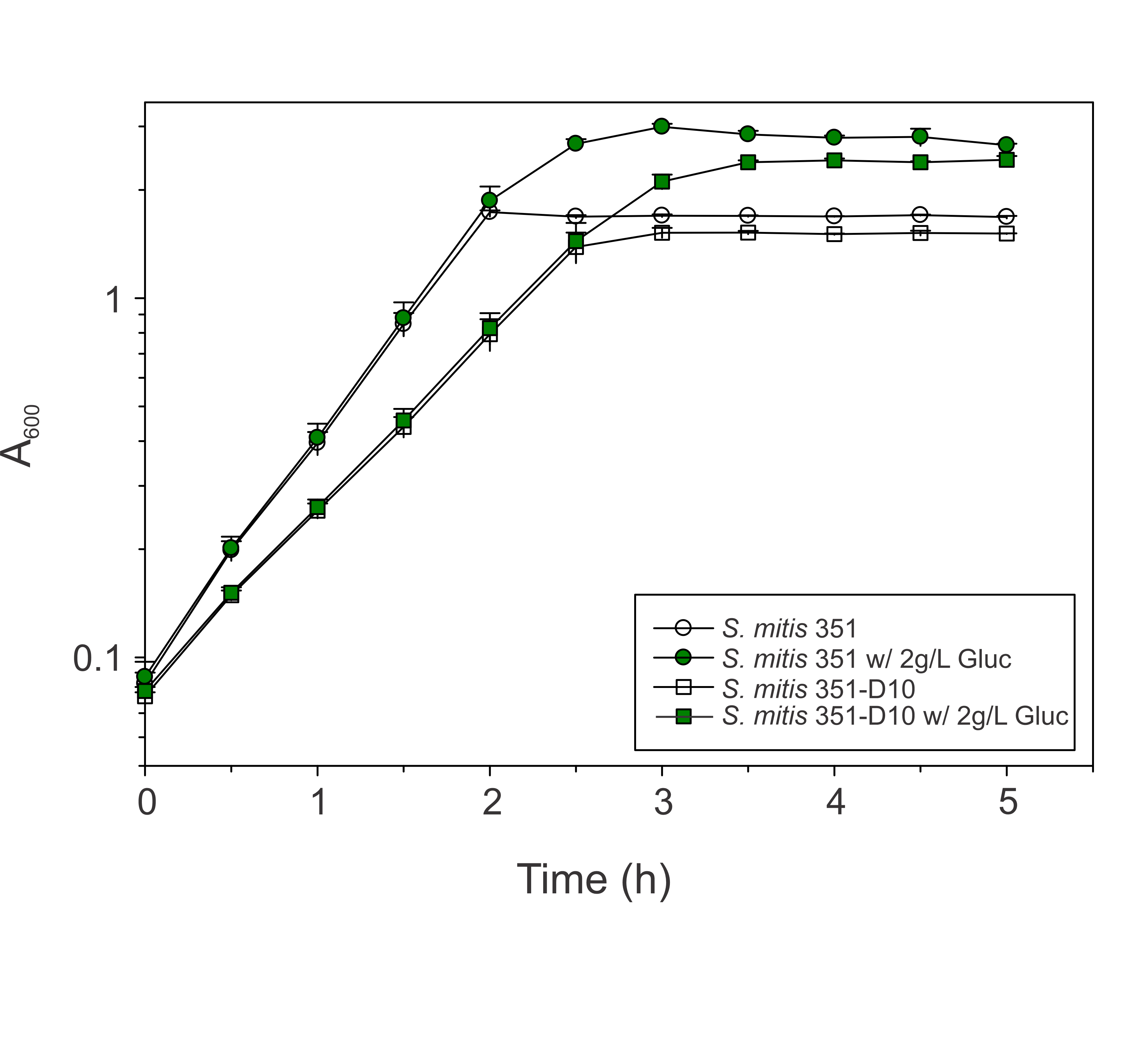

Supplement: Supplementary file 1 — Additional file 1: Figure S1. A semi-log plot of bacterial growth with supplemental glucose. [file 12866_2020_1849_MOESM1_ESM.tif]

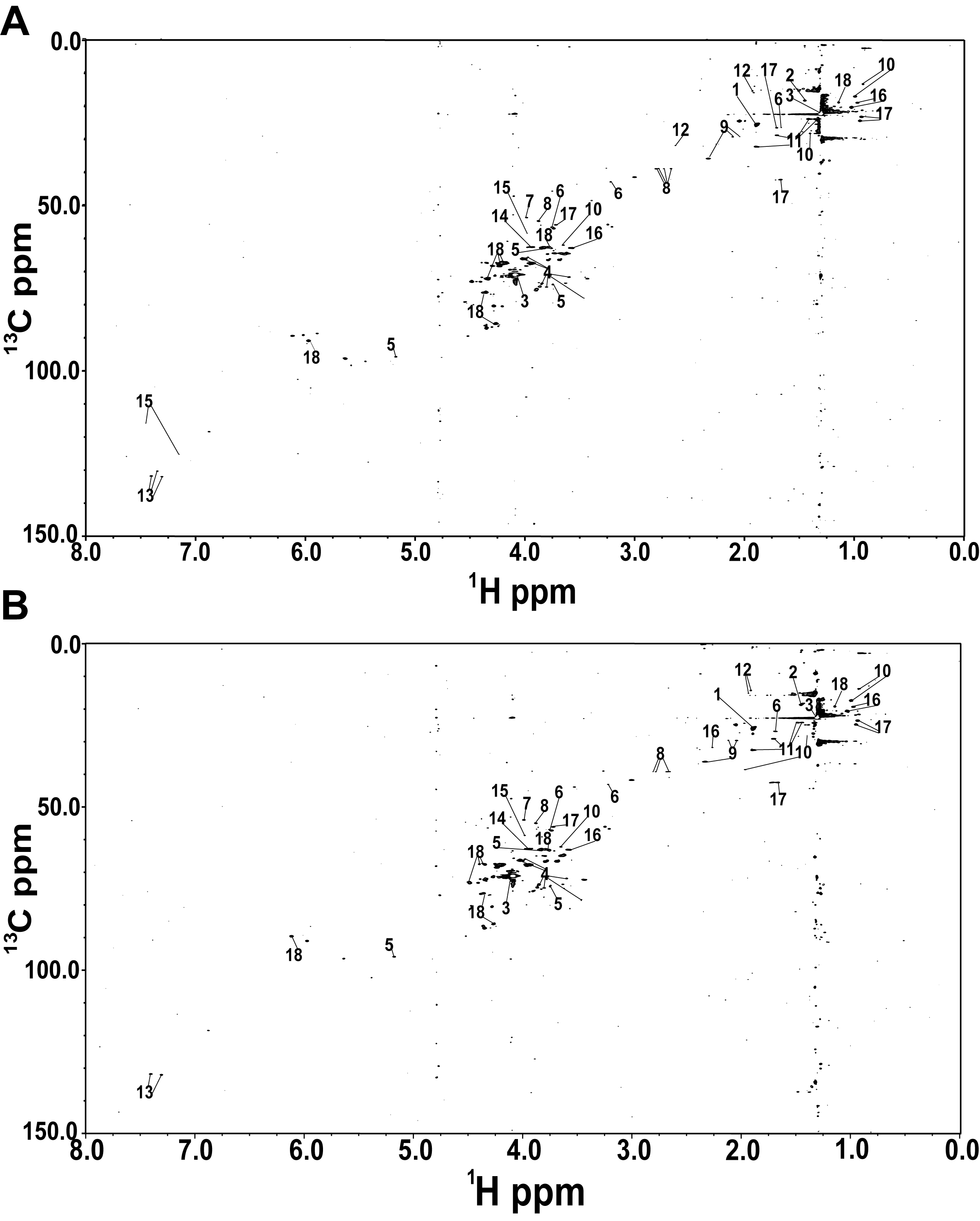

Supplement: Supplementary file 3 — Additional file 3: Figure S3. Representative 2D 1H 13C HSQC NMR spectra for S. mitis strains 351 and 351-D10 [file 12866_2020_1849_MOESM3_ESM.tif]
